# MARCH6 promotes hepatocellular carcinoma development through up-regulation of ATF2

**DOI:** 10.1186/s12885-021-08540-x

**Published:** 2021-07-17

**Authors:** Jie Sun, Zheng Dong, Zhengyao Chang, Hongfei Liu, Qiyu Jiang, Deyuan Zhang, Shanshan Lu, Xiaodong Jia, Dawei Wu, Aaron Ge, Pan Zhao, Jing Wang, Yinying Lu

**Affiliations:** 1 Comprehensive Liver Cancer Center, The Fifth Medical Center of PLA General Hospital, Beijing, 100039 China; 2 Department of General Surgery, The Fifth Medical Center of PLA General Hospital, Beijing, 100039 China; 3 Beijing Syngentech Co., Ltd., Beijing, 102206 China; 4 Research Center for Clinical and Translational Medicine, The Fifth Medical Center of PLA General Hospital, Beijing, 100039 China; 5 Department of Infectious Diseases, The Fifth Medical Center of PLA General Hospital, Beijing, 100039 China; 6 State key Laboratory of Toxicology and Medical Countermeasures, Beijing Institute of Pharmacology and Toxicology, Beijing, 100850 China

**Keywords:** Hepatocellular carcinoma, MARCH6, Proliferation, Migration, ATF2

## Abstract

**Background:**

Hepatocellular carcinoma (HCC) is a common cause of cancer mortality worldwide. Recent studies have shown that the polytopic enzyme membrane associated ring-CH-type finger 6 (MARCH6) participates in tumorigenesis, but its function in HCC development needs to be investigated. This study aimed to explore the role of MARCH6 in HCC.

**Methods:**

Expression of MARCH6 in human HCC samples was checked by immunohistochemical staining assay. Clinical relevance of MARCH6 and activating transcription factor 2 (ATF2) was analyzed from TCGA database. CCK-8, EdU staining, colony formation and transwell were performed to assess cell proliferation, growth and migration. Xenografted tumorigenesis was used to examine in vivo role MARCH6. Immunoblotting was applied to detect protein abundance.

**Results:**

We found that MARCH6 expression was elevated in human HCC samples. Over-expression of MARCH6 was associated with poor prognosis of HCC patients. Up-expression of MARCH6 promoted cell growth and migration of HCC cells. In contrast, the HCC cell growth and migration were suppressed by MARCH6 knockdown. Furthermore, the DNA synthesis was enhanced by MARCH6. The expression of ATF2 was potentiated by MARCH6 over-expression, while it was suppressed by MARCH6 silencing. TCGA database showed positive correlation between the expression of MARCH6 and ATF2. Importantly, ATF2 expression contributed to the oncogenic function of HCC cells.

**Conclusion:**

Our findings suggest that MARCH6-mediated ATF2 up-regulation contributes to HCC development. MARCH6 may be a promising target for the diagnosis and treatment of HCC.

**Supplementary Information:**

The online version contains supplementary material available at 10.1186/s12885-021-08540-x.

## Background

Hepatocellular carcinoma (HCC) is digestive malignancy with poor clinical outcomes and accounts for the second leading cause of cancer death worldwide [1, 2]. Over the past decade, major advances have been obtained to understand the epidemiologic risk factors as well as the molecular mechanisms that drive HCC tumorigenesis [1, 2]. As ubiquitination of proteins mediated proteolytic degradation in post-translational level, increasing amount of evidence indicates that protein ubiquitination is involved in a multitude of processes, including endocytosis, DNA repair, signal transduction and tumorigenesis [3–6].

Protein ubiquitination is an important post-transcriptional modification that regulates protein stability. With the aid of E3 ligases, ubiquitin is ligated to its specific substrate [7]. As a subfamily of the RING-type E3 ubiquitin ligase, membrane associated ring-CH-type finger (MARCH) proteins are discovered as the human homologs of the viral membrane ubiquitin ligases K3 and K5 from Kaposi’s sarcoma-associated herpesvirus [8]. Most MARCH family members have been indicated to be implicated in the development of human diseases [9, 10]. Among them, MARCH6 was first identified as a TEB4, which mediates the basal and cholesterol-induced degradation of squalene epoxidase [11]. The stability of MARCH6 was regulated by ubiquitin-specific protease 19 [12]. Recent studies indicated that MARCH6 was positively corrected with androgen receptor (AR) gene expression, which shared common regulation with MARCH6 by the transcription factor Sp1 in prostate cancer [13]. Additional studies suggested that MARCH6 and IDOL E3 ubiquitin ligase circuit played a multifaceted role in the regulation of cholesterol homeostasis in hepatocytes [14]. However, the specific functions of MARCH6 in HCC remain unclear.

Activating transcription factor 2 (ATF2) is a transcription factors which belongs to activator protein 1 (AP-1) family [15]. Aberrant expression or activation of ATF2 regulates the expression of a wide range of genes, which are involved in cell survival, proliferation, growth, apoptosis and DNA damage response, thereby playing an important role in cancer progression [16, 17]. For example, ATF2 functions as an oncogene in kinds of cancer types, including breast cancer [18], melanoma [19], lung cancer [20] and colorectal cancer [21]. Although it is well known that ATF2 contributes to carcinogenesis through regulating the expression of downstream targets, the upstream modulator of ATF2 and its significance in HCC remain to be determined.

Herein, we designed the present study to explore the role of MARCH6 in HCC. To this aim, we constructed MARCH6 knockdown and overexpressing HCC cells and examined HCC cell proliferation, migration and tumorigenesis. Our study not only identified MARCH6 as an oncogene, but also demonstrated ATF2 as an contributor of MARCH6 in HCC development.

## Methods

### MARCH6 expression analysis from TCGA database

MARCH6 transcripts in human HCC samples (tumor, *n* = 373; normal, *n* = 50) were analyzed from The Cancer Genome Atlas (TCGA). For overall and disease free survival analysis, HCC patients were subdivided into MARCH6 high expression group and low expression group. Group cutoff was set by quartile. The study was approved by the ethics committees of the Fifth Medical Center of Chinese PLA General Hospital.

### Immunohistochemical staining of MARCH6

10 pairs of HCC and adjacent normal tissues were used in this study. 5 um of paraffin-embedded tissues were subjected to immunohistochemical staining of MARCH6, following the indicated protocols as described previously [22, 23]. Quantification of MARCH6 protein abundance was determined by image J software.

### Cell culture

Huh7, HCCLM3 and MHCC97H cells were from Shanghai Zhong Qiao Xin Zhou Biotechnology Co.,Ltd. [24, 25]. Cells were cultured in DMEM (Invitrogen) in a 37 °C incubator with 5% CO_2_, supplied with 10% fetal bovine serum (Gibco) and 1% antibiotics (Corning).

### Lentivirus-mediated MARCH6 knockdown

Lentivirus vector pLL3.7 was used for MARCH6 knockdown in MHCC97H cells. The shRNA sequences were listed: shCtrl, 5′-TTCTCCGAACGTGTCACGT-3′; shMARCH6#1, 5′-GCACACTGTGTGCATTCATCA-3′; shMARCH6#2, 5′-GCTTGTGGTCTCTATGTTTGC-3′. shATF2, 5′-GGAGCCTTCTGTTGTAGAAAC-3′.

### MARCH6 over-expression

The CDS sequence of MARCH6 and ATF2 was constructed into pCDH lentivirus vector. Packaging vectors and pCDH vectors were transfected into 293 T cells. The virus were collected 48 h later and used to infect HCC cells.

### Cell proliferation

CCK-8 was applied to determine cell proliferation. Equal number of the indicated cells was seed into the 96-well plates, which contained 200 μl culture medium. At indicated time, 20 μl CCK-8 regent (Beyotime) was incubated with the culture medium at 37 °C for three hours. Cell viability was determined by measuring OD450 on a micro-plate reader.

### Colony formation

For MHCC97H cells, a total 10,000 shCtrl, shMARCH6–1 and shMARCH6–1 + ATF2 MHCC97H cells were plated. For Huh7 and HCCLM3 cells, a total 500 Ctrl, MARCH6 and MARCH6 + shATF2 Huh7 and HCCLM3 cells were plated. Colonies were grown for 10 days and then were stained by crystal violet (0.1%). The images were photographed by the camera.

### EdU staining

BeyoClick™ EdU Cell Proliferation Kit was applied to detect EdU cooperation. In brief, equal number of MHCC97H and Huh7 cells was seeded onto the coverslip in the 6-well plates. 16 h later, the cells were stained with EdU and DAPI, following the protocols.

### Transwell

Cell migration was determined by using transwell assay, following the protocols. For MHCC97H cells, a total of 10^6^ shCtrl, shMARCH6–1 and shMARCH6–1 + ATF2 MHCC97H cells were seeded per well. For Huh7 and HCCLM3 cells, a total of 5 × 10^5^ Ctrl, MARCH6 and MARCH6 + shATF2 Huh7 and HCCLM3 cells were seeded per well. 24 h later, cell migration was detected by staining with 0.1% crystal violet.

### Immunoblotting

Proteins were extracted using lysis buffer. Protein concentration was assessed by BCA method (Beyotime). Total proteins was subjected to sodium dodecyl sulfate-polyacrylamide gel electrophoresis (SDS-PAGE) and transferred to PVDF membranes (Millipore, USA), then blocked with 5% skim milk. After blocking, the membranes were incubated with primary antibodies followed by incubation with secondary antibodies. The signal was detected by chemiluminescence (Thermo Fisher Scientific). Primary antibodies against MARCH6 (ab183533) and ATF2 (ab47476) were purchased from Abcam. GAPDH (60004–1-Ig) antibody was purchased from Proteintech.

### Animal model

A total of 1 × 10^6^ MHCC97H cells expressing shCtrl or shMARCH6 lentivirus were subcutaneously implanted into BALB/c nude mice (female, 4–5-weeks old, randomly grouped for two groups, *n* = 5, 16–18 g per mice), which were purchased from Charles River (Beijing, China). The mice were kept under SFP room with 12-h light/12-h dark cycles. Tumor volume was monitored twice a week. The mice were anesthetized by pentobarbital sodium and were euthanized by carbon dioxide 37 days after implantation. Animal experiments were performed according the provisions of the Declaration of Helsinki and were approved by the Ethical Committee for Animal Experimentation of The Fifth Medical Center of PLA General Hospital.

### Statistical analysis

Statistical analysis was performed by SPSS version 16.0 software. Data were shown as the mean ± SEM of three independent experiments. Group comparisons were performed using t test or one-way ANOVA. Spearman correlation analysis was performed to study the correlation between MARCH6 and oncogenes in cancer growth and metastasis. Kaplan-Meier analysis of event or event-free rates was performed and statistical differences were evaluated by the log-rank test. Tests were two-sided and a probability (*P*) value of less than 0.05 was considered statistically significant.

## Results

### MARCH6 is highly expressed in human HCC cells

**Fig. 1:**
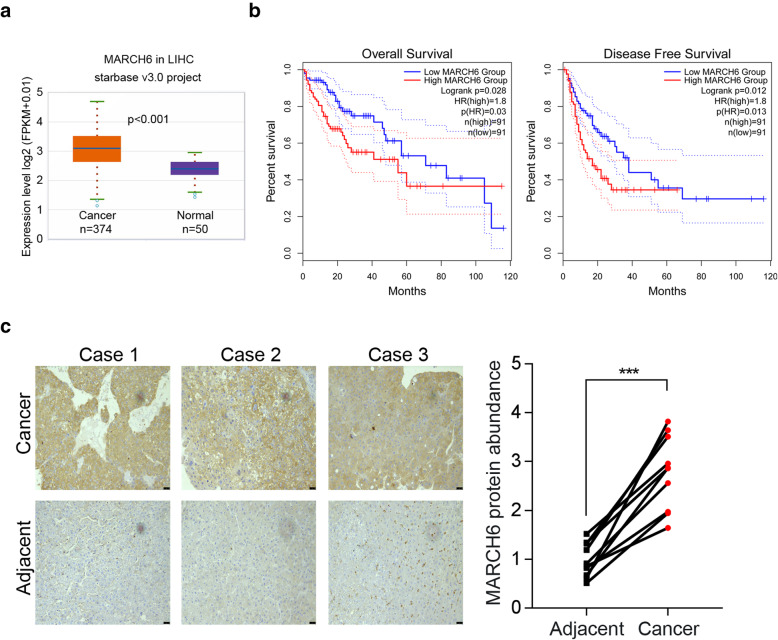
MARCH6 is over-expressed in HCC tissues. (**a**) TCGA database showed that MARCH6 was up-regulated in HCC samples. *P* < 0.001. (**b**) The overall and disease-free survival was determined in HCC patients who had MARCH6 high and low expression. Cutoff-High is 75% and Cutoff-Low is 25%. *n* = 91 for each group. *P* < 0.05. (**c**) Immunohistochemical analysis of MARCH6 in HCC and adjacent tissues. Scale bar, 25 um. *** *P* < 0.001

Firstly, we evaluated the clinical relevance of MARCH6 from TCGA database. We found that MARHC6 abundance was elevated in HCC samples (Fig. 1A). Furthermore, the overall survival and disease-free survival were both shorter in HCC patients who had MARCH6 high level compared to those who had MARCH6 low level (*P* < 0.05, Fig. 1B). Nevertheless, MARCH6 expression was not significantly associated with HCC patients’ T stage and grade (Table 1). Then, we performed immunohistochemical staining of MARCH6. As shown in Fig. 1C, MARCH6 was up-regulated in HCC tissues compared to adjacent normal samples. Furthermore, Spearman correlation analysis in TCGA HCC tissues revealed that MARCH6 was positively correlated with MKI67, CTNNB1, CDH2, FN1 and WNTSA, which were demonstrated as oncogene in cancer growth and metastasis (Supplementary Fig. 1). By contrast, MARCH6 was negatively associated with BAD and BAX, which played an important role in apoptosis (Supplementary Fig. 1). Taken together, MARCH6 was a potential oncogene for HCC.

**Table 1:** Correlation between patients’ characteristics and MARCH6 expression in LIHC from TCGA database

Characteristics		MARCH6		Total	*P* value
		High	Low		
Sex	Male	124	126	250	.883
	Female	61	60	121	
Total		185	186	371	
Age	≤65	124	108	232	.085
	> 65	61	77	138	
Total		185	185	370	
T stage	T1/2	134	141	175	.401
	T3/4	50	43	93	
Total		184	184	368	
Grade	G1/2	108	124	232	.083
	G3/4	75	59	134	
Total		183	183	366	

### MARCH6 increases the proliferation, growth and DNA synthesis of HCC cells

**Fig. 2:**
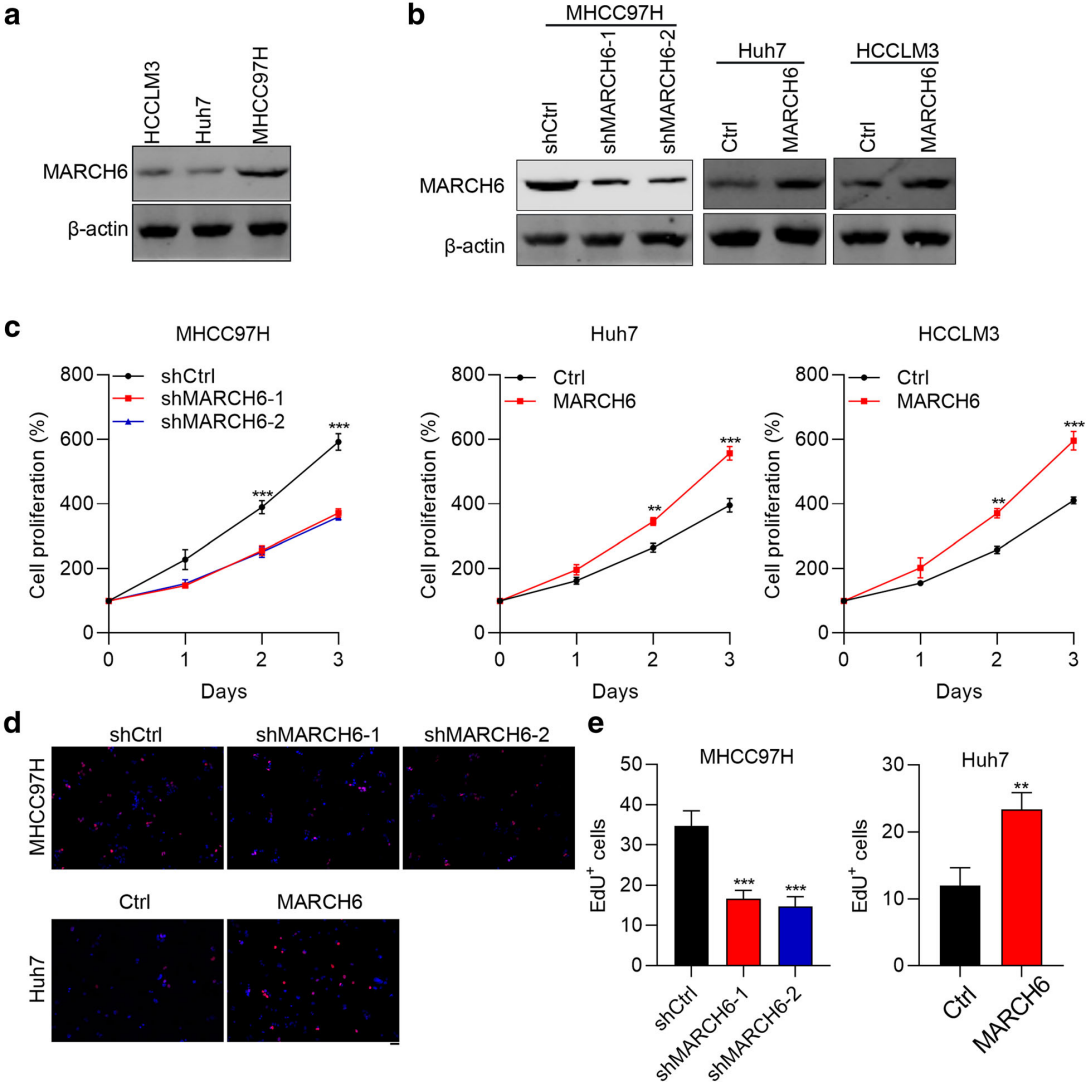
MARCH6 expression is essential for HCC cell growth. (**a**) Immunoblotting analysis of MARCH6 in HCCLM3, Huh7 and MHCC97H cells. (**b**) Immunoblotting analysis of MARCH6 in shCtrl, shMARCH6–1 and shMARCH6–2 MHCC97H cells, and in Ctrl and MARCH6 overexpressed Huh7 and HCCLM3 cells. (**c**) CCK-8 determined cell viability in shCtrl, shMARCH6–1 and shMARCH6–2 MHCC97H cells, and in Ctrl and MARCH6 overexpressed Huh7 and HCCLM3 cells. *n* = 3. (**d** and **e**) shCtrl, shMARCH6–1 and shMARCH6–2 MHCC97H cells, Ctrl and MARCH6 overexpressed Huh7 were subjected to EdU staining. *n =* 3. Scale bar, 50 um. ** *P* < 0.01, *** *P* < 0.001

To study the function of MARCH6, we firstly checked the expression of MARCH6 in three HCC cell lines, including Huh7, HCCLM3 and MHCC97H. The expression of MARCH6 was highest in MHCC97H cells as compared with Huh7 and HCCLM3 cells (Fig. 2A). We therefore infected MHCC97H cells with MARCH6 knockdown lentivirus and Huh7 or HCCLM3 cells with MARCH6 overexpressing lentivirus. Immunoblotting results showed that MARCH6 was efficiently knocked down in MHCC97H cells and was efficiently overexpressed in Huh7 and HCCLM3 cells (Fig. 2B). CCK assay demonstrated that MARCH6 silencing led to reduced cell viability of MHCC97H cells (Fig. 2C). By contrast, MARCH6 overexpression promoted the proliferation of Huh7 and HCCLM3 cells (Fig. 2D). DNA synthesis is a biomarker for cell proliferation. We performed EdU staining in HCC cells and found that the number of EdU positive cells was reduced but increased in MARCH6 knockdown and over-expressed HCC cells (Fig. 2D and E), respectively. Collectively, MARCH6 has oncogenic function in HCC cells.

### MARCH6 silencing retards the tumor growth of HCC cells

**Fig. 3:**
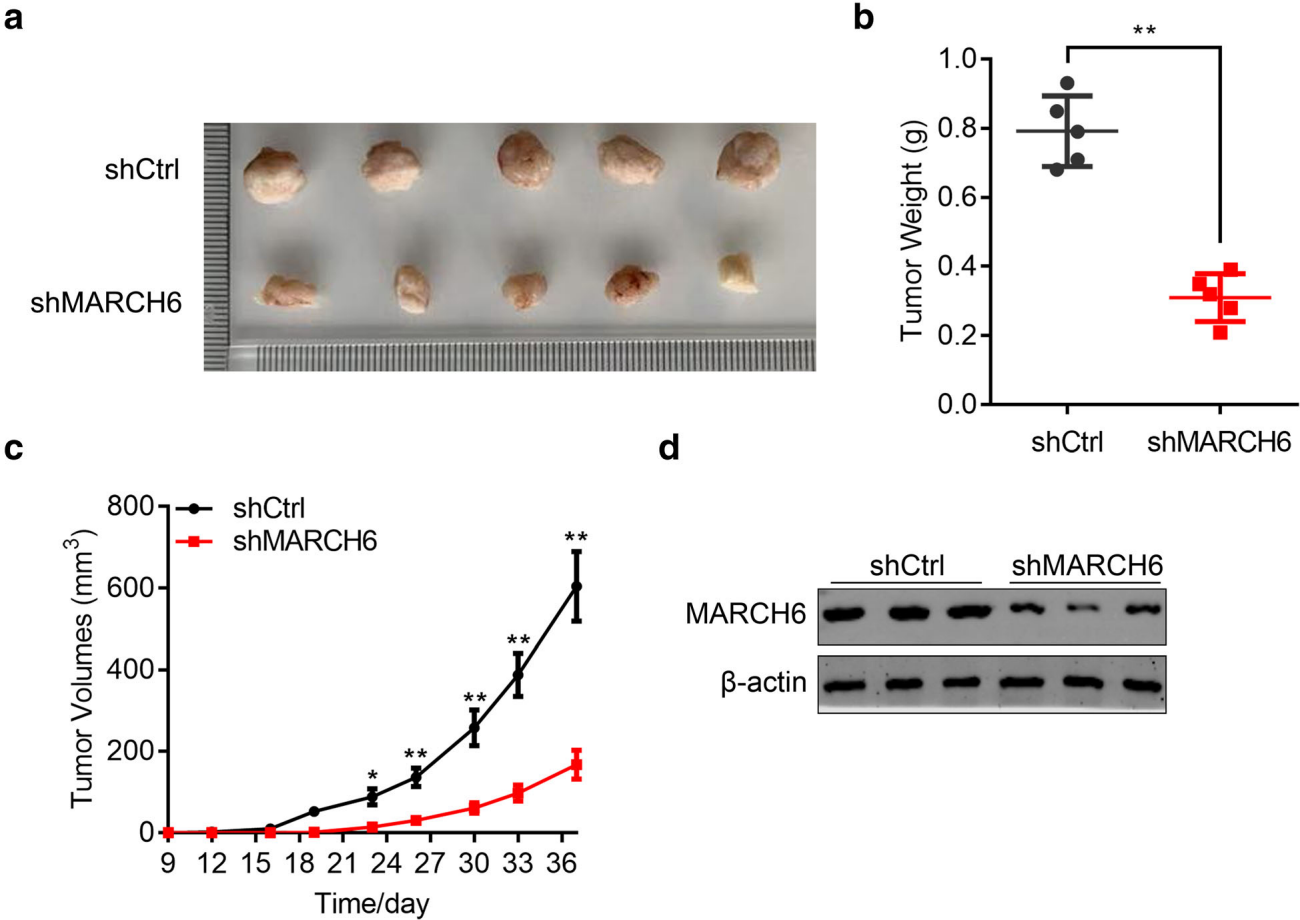
MARCH6 knockdown suppressed the xenografted tumorigenesis of HCC cells. (**a**) The images of xenografted tumors derived from shCtrl and shMARCH6 MHCC97H cells. *n =* 5 per group. (**b**) Tumor weight of xenografted tumors as shown in A. (**c**) Tumor volume of xenografted tumors as shown in A. (**d**) Immunoblotting analysis of MARCH6 in shCtrl and shMARCH6 tumor tissues. * *P* < 0.05, ** *P* < 0.01

To study the effect of MARCH6 silencing on tumorigenesis, shCtrl or shMARCH6 MHCC97H cells were subcutaneously transplanted into the right arms of nude mice (*n* = 5 per group). The results showed that MARCH6 knockdown in MHCC97H cells inhibited the tumorigenesis and growth (Fig. 3). Thus, MARCH6 is critical for HCC growth.

### MARCH6 up-regulates ATF2 in HCC cells

**Fig. 4:**
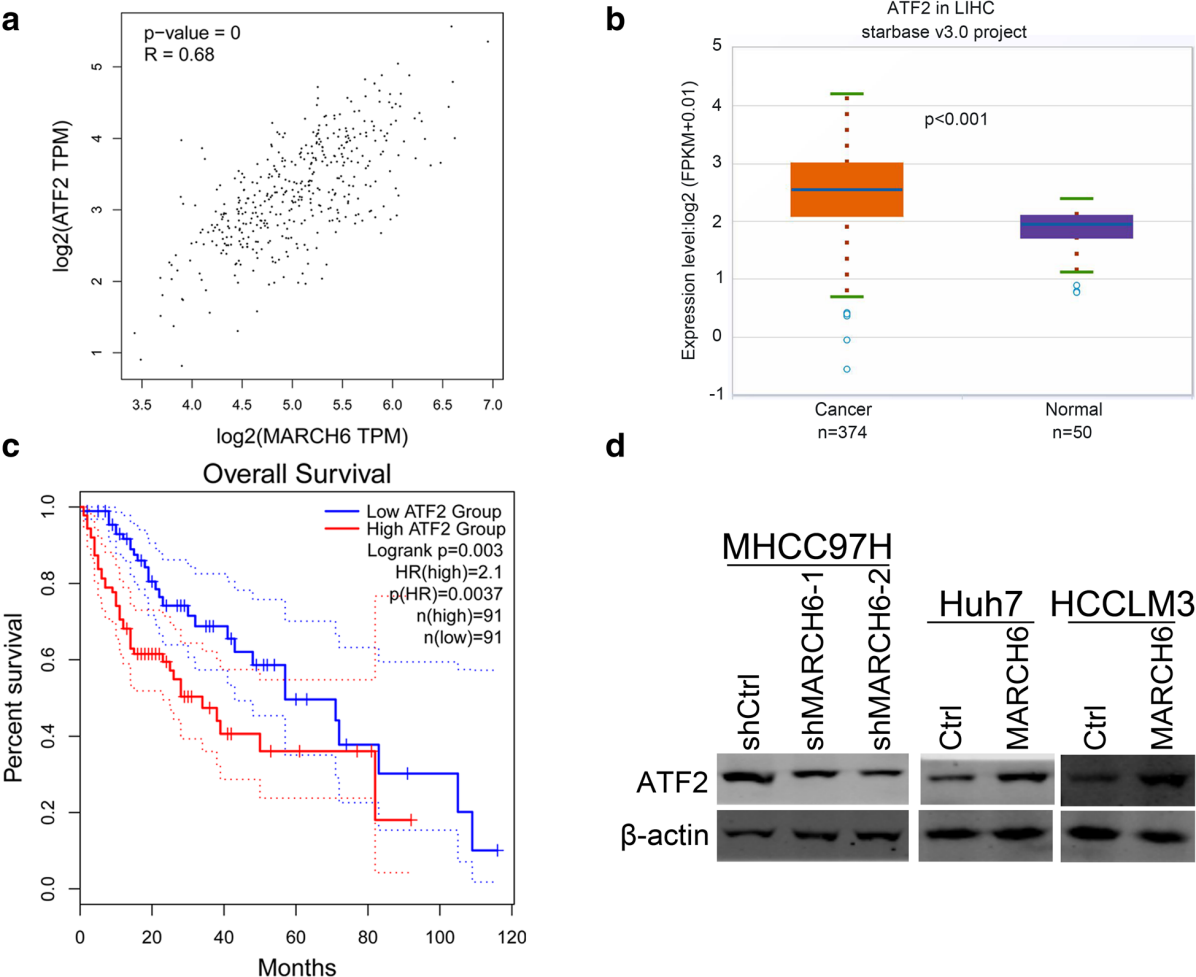
MARCH6 up-regulates ATF2 in HCC cells. (**a**) Immunoblotting analysis of ATF2 in shCtrl, shMARCH6–1 and shMARCH6–2 MHCC97H cells, and in Ctrl and MARCH6 overexpressed Huh7 and HCCLM3 cells. (**b**) TCGA database showed that ATF2 was up-regulated in HCC tissues. *P* < 0.001. (**c**) TCGA database showed that ATF2 expression was inversely correlated with the survival of HCC patients. *P* = 0.003. (**d**) ATF2 expression was positively correlated with MARCH6. *P* < 0.001

To investigate the molecular mechanism underlying how MARCH6 promotes HCC, we analyzed the most correlated mRNAs from TCGA database. Among the database, we found that ATF2 was highly associated with MARCH6 expression (Fig. 4A). And the transcription level of ATF2 in HCC tumor sample was higher than the normal tissues (Fig. 4B). In addition, ATF2 expression level was inversely correlated with the survival of HCC patients (Fig. 4C). We then checked the expression of ATF2 in MARCH6 knockdown and over-expressed cells. Protein expression of ATF2 was reduced and elevated in MARCH6 silenced and over-expressed HCC cells, respectively (Fig. 4D). These results suggest that MARCH6 could up-regulate ATF2 in HCC cells.

### Over-expression of ATF2 restores the growth and migration of MHCC97H cells with down-regulation of MARCH6

**Fig. 5:**
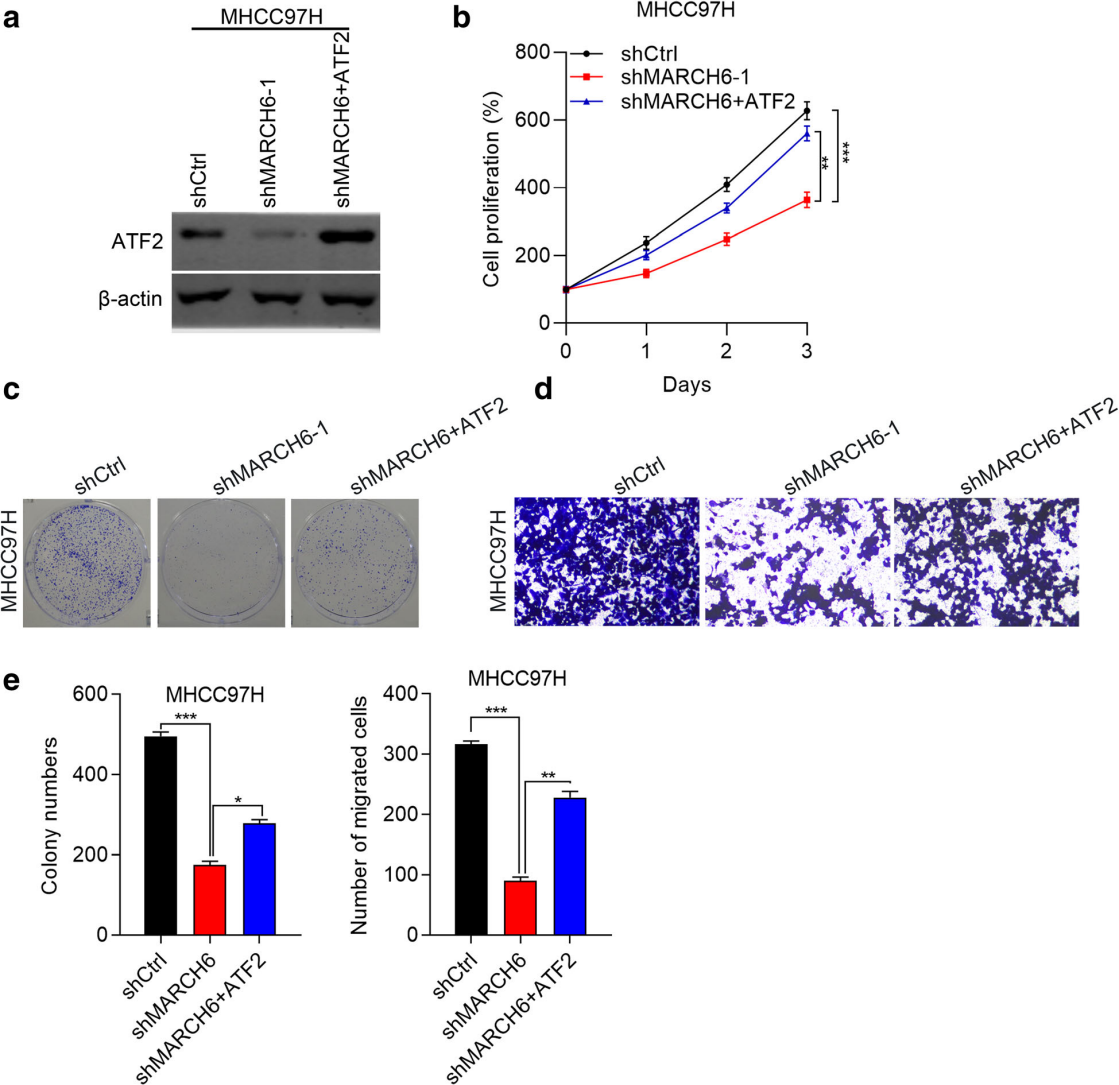
Over-expression of ATF2 restores the growth and migration of MHCC97H cells with down-regulation of MARCH6. (**a**) Immunoblotting analysis of ATF2 in shCtrl, shMARCH6–1 and shMARCH6–1 + ATF2 MHCC97H cells. (**b** and **c**) Cell proliferation was checked by CCK-8 assay (**b**) and colony formation was determined (**c**) in shCtrl, shMARCH6–1 and shMARCH6–1 + ATF2 MHCC97H cells. (**d**) Cell migration was determined by transwell assay in shCtrl, shMARCH6–1 and shMARCH6–1 + ATF2 MHCC97H cells. (**e**) Quantification results of colony formation and cell migration. * *P* < 0.05, ** *P* < 0.01, *** *P* < 0.001

To explore whether MARCH6 knockdown suppresses the growth and migration of HCC cells through regulating the expression of ATF2, we infected MARCH6 silenced MHCC97H cells with ATF2 over-expressing lentivirus. As shown in Fig. 5A, ATF2 was efficiently over-expressed in MARCH6 silenced MHCC97H cells. CCK8 and colony formation results demonstrated that MARCH6 knockdown suppressed the proliferation and colony growth of MHCC97H cells, which could be restored by ectopic expression of ATF2 (Fig. 5B and C). ATF2 over-expression also restored the migration capacity of MHCC97H cells with silencing of MARCH6 (Fig. 5D). Our results indicate that MARCH6 up-regulation of ATF2 contributes to HCC cell growth and migration.

### Knockdown of ATF2 reverses the growth and migration of HCC cells with over-expression of MARCH6

**Fig. 6:**
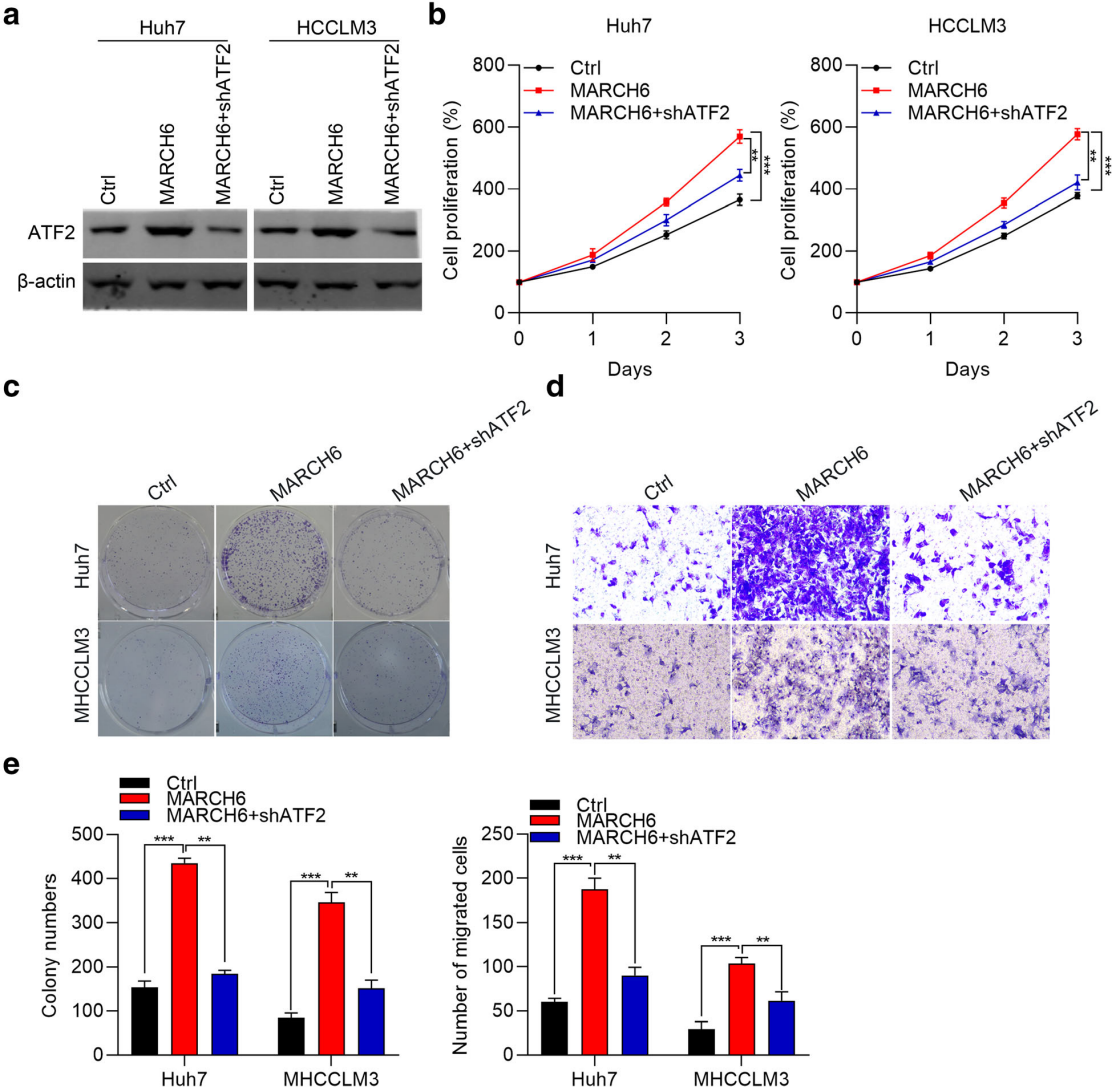
Knockdown of ATF2 reverses the growth and migration of HCC cells with over-expression of MARCH6. (**a**) Immunoblotting analysis of ATF2 in Ctrl, MARCH6 and shMARCH6 + shATF2 Huh7 and HCCLM3 cells. (**b** and **c**) Cell proliferation was checked by CCK-8 assay (**b**) and colony formation was determined (**c**) in Ctrl, MARCH6 and shMARCH6 + shATF2 Huh7 and HCCLM3 cells. (**d**) Cell migration was determined by transwell assay in Ctrl, MARCH6 and shMARCH6 + shATF2 Huh7 and HCCLM3 cells. (**e**) Quantification results of colony formation and cell migration. ** *P* < 0.01, *** *P* < 0.001

To further confirm the role of MARCH6/ATF2 axis in HCC cell growth and migration, we infected MARCH6 over-expressing Huh7 and HCCLM3 cells with ATF2 knockdown lentivirus. When MARCH6 over-expression promoted the expression of ATF2, knockdown lentivirus could significantly down-regulate ATF2 (Fig. 6A). ATF2 knockdown significantly reduced the proliferation and migration ability of Huh7 and HCCLM3 cells with over-expression of MARCH6 (Fig. 6B-E). Consistently, ATF2 down-regulation reversed the promoting role of MARCH6 up-regulation on the migration ability of Huh7 and HCCLM3 cells (Fig. 6D and E). Collectively, MARCH6/ATF2 axis is essential for HCC cell growth and migration.

## Discussion

HCC is a common cause of cancer mortality [26]. Current data indicate that the emerging trends in HCC incidence is steadily increasing worldwide [27]. Till now, surgical resection, radiofrequency ablation and liver transplantation remain the widely used options for HCC patients, but with unsatisfactory clinical outcome due to a high risk of recurrence [28]. Studies have reported that the carcinogenesis and pathogenesis of HCC are involving in a wide variety of factors [29]. Notably, ubiquitination serves as a degradation mechanism of proteins, involved in a series of cellular processes, such as DNA damage repair, immune response and tumor metastasis, which is supported by multiple studies [30]. Among numbers of molecules and signal pathways attributed to protein ubiquitination, MARCH6 was suggested to function as a key player in modulating inflammatory response, lipoprotein uptake and tumor cell proliferation [14, 31, 32]. Despite that MARCH6 is an important E3 ligase, the specific functions of MARCH6 in HCC are poorly understood. Moreover, its mechanisms underlying the oncogene properties of MARCH6 in HCC were unexplored.

The expression level of MARCH6 is up-regulated in and knockdown of MARCH6 would inhibit the capacity of proliferation in breast cancer cells in previous study [31], but the expression pattern of MARCH6 in HCC remains unclear. Thus, we firstly examined MARCH6 expression and identified a strong correlation between MARCH6 expression and HCC patient’s overall survival rate from clinical samples and public TCGA database. In addition, we presented evidence that MARCH6 knockdown attenuated the proliferative, colon formation capacity and DNA synthesis of HCC cell lines, as well as the ability of migration. Opposite results were obtained along with over-expression of MARCH6. Furthermore, to uncover the molecular mechanism responsible for the important function of MARCH6, we analyzed the most correlated mRNAs from TCGA database and found that ATF2 is highly associated with the expression level of MARCH6, which suggested MARCH6 up-regulates ATF2 in HCC cells. Also we examined the significance of ATF2 and found ATF2 potentiates HCC cell function. Nevertheless, the detailed interaction between MARCH6 and ATF2 in the progression and development of HCC remain largely elusive.

There are some limitations in this study. For example, how MARCH6 regulated ATF2 was unclear. MARCH6 is an E3 ligase which degrades the abundance of downstream substrate via interaction. However, MARCH6 potentiates ATF2 at both mRNA and protein level, suggesting that MARCH6 may not interact with ATF2. Additionally, whether MARCH6 interacts with and regulates the upstream factors of ATF2 needs further studies.

## Conclusion

In conclusion, our study showed that MARCH6 was up-regulated in HCC. In addition, MARCH6 overexpression promoted the proliferative and invasive ability of HCC cells by potentiating the expression of ATF2, suggesting that ectopic MARCH6 expression may be an important driver for HCC development. MARCH6 may be a promising target for the diagnosis and treatment of HCC.

## Supplementary Information

**Additional file 1: Supplementary Fig. 1**. MARCH6 is negatively associated with pro-apoptotic proteins.

## Data Availability

The data generated in this study are included in this published article.

## Abbreviations

HCC: Hepatocellular carcinoma
MARCH6: Membrane associated ring-CH-type finger 6
ATF2: Activating transcription factor 2
AR: Androgen receptor
TCGA: The Cancer Genome Atlas
SDS-PAGE: Sodium dodecyl sulfate-polyacrylamide gel electrophoresis
