# Supplementary material for: MARCH6 promotes hepatocellular carcinoma development through up-regulation of ATF2
Source: BMC Cancer. 2021 Jul 17;21:827. doi: 10.1186/s12885-021-08540-x (PMC8285810; doi:10.1186/s12885-021-08540-x)

Supplementary Figure 1

The correlation between MARCH6 and MKI67, BAD, BAX, CTNNB1, CDH2, FN1 and WNT5A was analyzed from TCGA database. r and p value was shown as indicated.


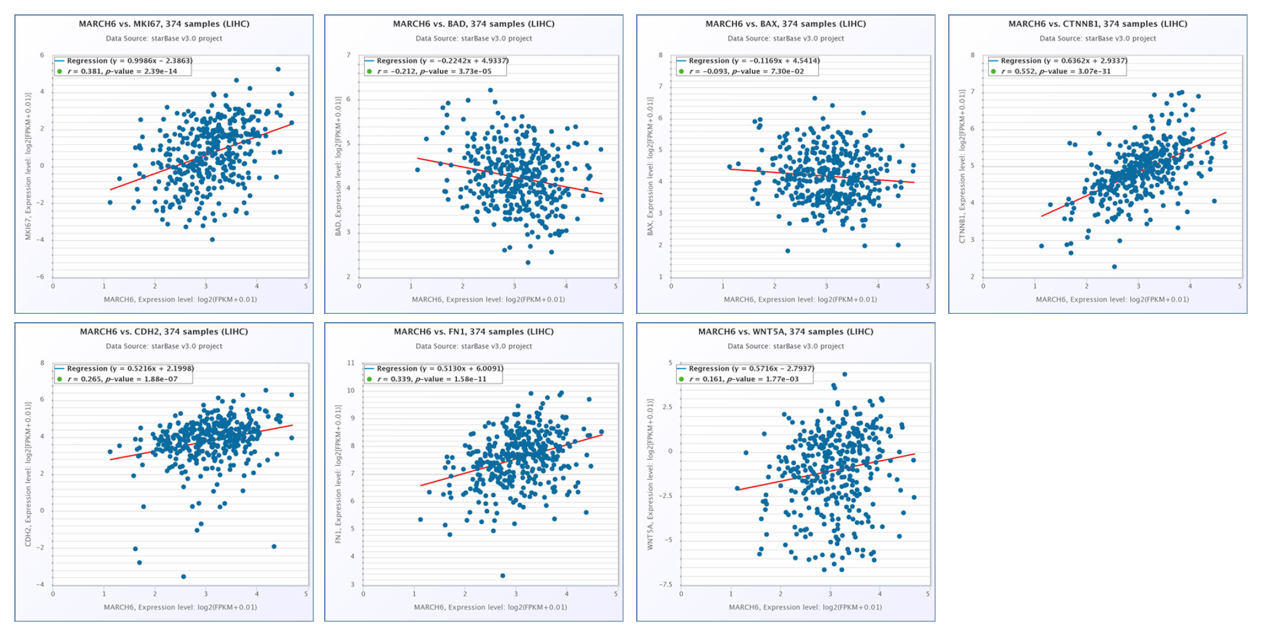


Fig. 1 Full length immunoblotting images.


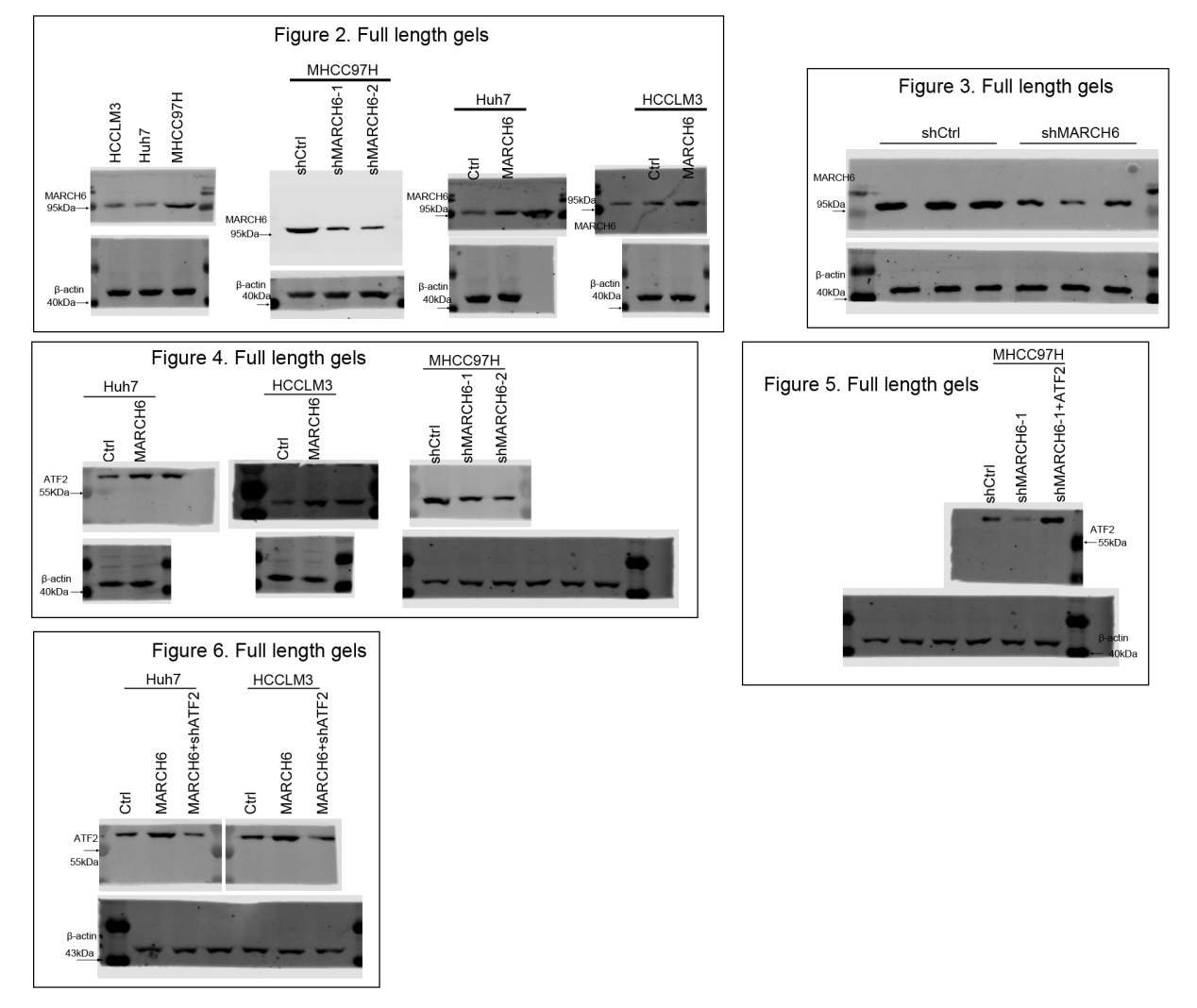

Supplement: Supplementary file 1 — Additional file 1: Supplementary Fig. 1. MARCH6 is negatively associated with pro-apoptotic proteins. [file 12885_2021_8540_MOESM1_ESM.docx]
